# Prevalence and correlates of sexual dysfunction among patients with mental disorders in a tertiary hospital in Southwest Nigeria

**DOI:** 10.4102/sajpsychiatry.v27i0.1575

**Published:** 2021-05-27

**Authors:** Adekunle O. Adesola, Bibilola Oladeji

**Affiliations:** 1 Department of Psychiatry, Faculty of Clinical Sciences, University College Hospital, Ibadan, Nigeria

**Keywords:** prevalence, correlates, sexual dysfunctions, mental disorders, Southwest Nigeria

## Abstract

**Background:**

Sexual dysfunction is more common among patients with mental disorders compared to the general population. Despite this high occurrence, information regarding sexual dysfunction as well as their correlates in patients receiving care for mental disorders in developing countries is still sparse.

**Aim:**

To determine the prevalence and correlates of sexual dysfunction among patients with mental disorders receiving care in a tertiary hospital in Southwest Nigeria.

**Setting:**

This study was performed at the psychiatric outpatient clinic at the University College Hospital, Ibadan, Southwest Nigeria.

**Methods:**

A cross-sectional study was conducted on a convenience sample of 238 adults aged 18–60 years. Socio-demographic and clinical information was obtained from all recruited patients. Sexual dysfunction was assessed using the International Index of Erectile Function questionnaire for men and the Female Sexual Function Index questionnaire for women. Questionnaires for measuring depression, medication adherence and autonomic medication side effects were also administered. Associations between sexual dysfunction and socio-demographic and clinical factors were explored.

**Results:**

The prevalence rates of sexual dysfunction among male and female participants were 84.7% and 95.7%, respectively. In the multivariate analysis, employment status and autonomic side effects of psychotropic medications significantly predicted male sexual dysfunction, while religion and employment status were predictors of female sexual dysfunction.

**Conclusion:**

Sexual dysfunction is very common among patients with mental disorders, with higher rates in female participants. There is a need for clinicians to consider routine screening for sexual dysfunction in psychiatric outpatients with a view of providing psychosocial interventions to improve patient’s quality of life.

## Introduction

Sexual activity is regarded by the World Health Organization and the Office of Surgeon General of the United States as a basic human right and an integral part of life.^1^ Disturbance in sexual functioning is known to affect individuals’ sense of personal satisfaction and impair health-related quality of life (QoL).^2^ Sexual dysfunction is quite common, with rates ranging between 30% and 80.8% in the general population.^3,4,5,6,7,8^ In a study among female students in a university in Nigeria, Nwagha et al. reported a rate of 53.3%.^4^

Sexual dysfunction is reportedly higher in patients with mental disorders compared to the general population, with rates ranging between 40% and 96%.^9,10,11,12,13,14,15,16,17,18,19^ Studies reveal that 40% – 90% of patients with schizophrenia have one or more forms of sexual dysfunction.^9,10,12^ Among patients with depression on treatment, sexual dysfunction is present in up to 50% of the female participants and 40% of the male participants.^13^ In a recent study conducted in Nigeria, Olisah et al. found a prevalence rate of 64.3% among outpatients with schizophrenia, substance use disorder, recurrent depressive disorder and bipolar affective disorder receiving psychotropic medications.^14^

Studies on the rates of sexual dysfunction types among patients with mental disorders and those without have not consistently demonstrated a difference. While some studies report that sexual dysfunction prevalence is higher in patients with mental disorders,^20^ others show lower prevalence rates.^14^ According to a study conducted by Simiyon et al. among Indian women with schizophrenia, the rates of sexual desire, arousal and orgasmic disorders were reported to be 100%, 92.1% and 76.2%, respectively.^20^ In contrast, Esfehani et al. reported lower rates of 49.2%, 43.2% and 38.6% for sexual desire, arousal and orgasmic disorders, respectively, among Iranian women in the general population.^21^ Most studies on the prevalence of sexual dysfunction among men focussed on erectile dysfunction and also showed an inconsistent pattern. Although Oyelade et al. reported a prevalence rate of 58.9% among men in the general population,^22^ Olisah et al. reported a lower prevalence of 40.2%,^14^ while Mosaku et al. reported a higher prevalence of 83%^15^ among male patients with mental disorders.

Factors that are associated with sexual dysfunction among patients with mental disorders include increasing age, female sex, marital status, quality of marital relationship, employment status, use of first-generation antipsychotics such as haloperidol, medication dosage, medication side effects, comorbid medical conditions and presence of psychopathology.^11,12,14,15,20^ Psychotropic medications and their adverse effects are important risk factors for sexual dysfunction in patients with mental disorders. In a study conducted in the United Kingdom, 45% of patients on antipsychotic medications had sexual dysfunctions compared to 17% of normal controls.^18^ In patients on antidepressants, sexual dysfunction ranged between 25.8% and 80.3%.^23^ This impaired ability in sexual experience and performance not only adds to the suffering and disability associated with mental disorders, but also increases the likelihood of non-adherence to treatment, and contributes to diminished self-esteem and confidence and negatively affects QoL.^13^

The few available studies on sexual dysfunction among patients with mental disorders conducted in Nigeria have estimated prevalence rates ranging from 40.4% to 83%.^12,14,15^ None of these studies explored the relationship between sexual dysfunction and adherence to psychotropic medications. It is also not clear from the available studies whether there are associations between sexual dysfunction and depressive symptoms as well as autonomic side effects of psychotropic medications among patients with mental disorders in Nigeria. This study was designed to fill some of these gaps by providing information on the prevalence and correlates of sexual dysfunction in patients attending a psychiatric clinic in Nigeria. Specifically, we explored the relationship between sexual dysfunction and medication adherence, depressive symptoms and autonomic side effects of psychotropic medications.

## Methods

This study followed a descriptive, cross-sectional study design. Participants were consecutive consenting patients attending the psychiatric clinic at the medical outpatient department of the University College Hospital, Ibadan over a period of 6 months from November 2016 to April 2017. The University College Hospital is a tertiary teaching hospital in Southwestern part of Nigeria.

The sample size for this study was determined using the sample size formula for cross-sectional studies. An estimated sample size of 240 was derived based on the 83% prevalence of sexual dysfunction among patients with mental disorders in a previous Nigerian study,^15^ adding a non-response rate of 10%.

Participants were adults (aged between 18 and 60 years), with a diagnosis of a mental disorder according to the International Classification of Diseases 10th edition and currently in partial or full remission, who provided voluntary written informed consent. Those who had acute illnesses, grossly disorganised thought or behaviour and/or significant cognitive impairment were excluded from the study.

Eligible participants were either given the questionnaires to complete by themselves with the support from two trained research assistants or had the questionnaires administered to them if they were unable to read or write English or Yoruba languages fluently.

### Instruments

Socio-demographic and clinical information were obtained from all recruited patients by self-report. Sexual dysfunction was assessed using the International Index of Erectile Function (IIEF) questionnaire for male participants and the Female Sexual Function Index (FSFI) questionnaire for female participants. Information on depressive symptoms, medication adherence and autonomic side effects of psychotropic medications were obtained using the Becks Depression Inventory (BDI), Morisky Medication Adherence Scale 8 (MMAS 8) and the autonomic subscale of the Udvalg for Kliniske Undersogelser Side Effects Rating Scale (UKU-SERS), respectively. The Yoruba versions of the questionnaires were derived by standard iterative procedures of translation and back-translation. Either the Yoruba or the English version was administered depending on the language the patients were more comfortable with.

#### International Index of Erectile Function Questionnaire

The IIEF is a brief, self-administered questionnaire used for the assessment of male sexual functioning in the past 4 weeks. It has five domains comprising 15 questions that are scored on a scale of 0–5, with 0 indicating no sexual activity or no attempt. These domains are erectile function (Q1–5, Q15) with a maximum score of 30, intercourse satisfaction (Q6–8) with a maximum score of 15, orgasmic function (Q9, 10) with a maximum score of 10, sexual desire (Q11, 12) with a maximum score of 10 and overall satisfaction (Q13, 14) with a maximum score of 10. Higher scores denote better sexual functioning. Based on the presence and severity of sexual dysfunction, each domain is further classified into ‘no dysfunction’, ‘mild dysfunction’, ‘mild to moderate dysfunction’, ‘moderate dysfunction’ and ‘severe dysfunction’. In this study, sexual dysfunction was defined as the presence of at least a mild dysfunction in one or more domains, as reported in the previous studies.^12,14^ In an earlier study in Nigeria,^15^ a reliability coefficient (Cronbach’s alpha) of 0.921 was obtained.

#### Female Sexual Function Index questionnaire

The FSFI is a brief, self-administered questionnaire used for the assessment of female sexual functioning in the past 4 weeks. It has six domains comprising 19 questions that are scored on a scale of 0–5, with 0 indicating no sexual activity or no attempt. These domains are sexual desire (Q1, 2), sexual arousal (Q3–6), lubrication (Q7–10), orgasm (Q11–13), satisfaction (Q14–16) and pain (Q17–19), which all have a maximum score of 6. The overall score ranges from a minimum score of 2 to a maximum score of 36, with a higher score connoting better sexual functioning. As proposed by Wiegel et al., a cut-off point of 26.55 or less was classified as female sexual dysfunction in this study.^24^ The instrument has been validated and shown to be sensitive and reliable in Nigeria, with Cronbach’s alpha of 0.8.^4^

#### Morisky Medication Adherence Scale 8

The MMAS 8 is an eight-item, self-administered questionnaire used for the assessment of medication adherence among patients with different clinical conditions and has been used in some Nigerian studies.^25,26^ Each item measures specific medication-taking behaviour and provides a measure adherence to medications. Items 1–7 were recorded as a ‘yes’ or ‘no’ dichotomous response and the last item was recorded using a five-point Likert scale. Morisky Medication Adherence Scale 8 scores can range from 0 to 8 and score ranges are classified into three levels of adherence to facilitate use in clinical practice: high adherence, a score of 0; medium adherence, a score of 1–2; and low adherence, a score of ≥ 3.

#### The Udvalg for Kliniske Undersogelser Side Effects Rating Scale

The UKU-SERS is a questionnaire that measures the side effects of medications in the past 3 days. It comprises 48 items that are categorised into psychic subscale (1.1–1.10), neurologic subscale (2.1–2.8), autonomic subscale (3.1–3.11) and others (4.1–4.19). The subscales are useful in assessing differential side effect profiles. The autonomic subscale of the self-rated version was used in this study to assess the presence of autonomic side effects of prescribed psychotropic medications. Each item is scored on a four-point Likert-type scale (0–3), a score of 0 indicating ‘not or doubtfully present’. Scores 1–3 indicate that a symptom is present to a mild, moderate or severe degree, respectively. The scale can be used in both clinical practice and trial. It has been adapted to suit the study population.

#### The Beck Depression Inventory

The BDI is a 21-item self-rated questionnaire designed to assess the intensity of depressive symptoms among individuals. Each item is scored on a Likert scale of 0–3 in terms of increasing intensity and is summed up giving a minimum and maximum score of 0 and 63, respectively. The total scores are interpreted as 0–10 (normal), 11–16 (mild mood disturbance), 17–20 (borderline depression), 21–30 (moderate depression), 31–40 (severe depression) and *˃* 40 (extreme depression). The BDI has been validated among the adolescent^27^ and adult population^28^ in Nigeria and has been widely used in previous Nigerian studies.^29,30^

## Statistical analysis

Data were cleaned and entered into Microsoft Excel spreadsheet and statistical analysis was performed using SPSS 22.0. Categorical data were summarised into tables, while continuous data were summarised using mean and standard deviation. Pearson’s chi-square test or Fisher’s exact test was used to determine the association between categorical dependent variables, while independent *t*-test or analysis of variance was applied to continuous dependent variables. Pearson’s correlation coefficient was used to test the association between continuous dependent and independent variables. The independent variables found to be significantly associated with sexual dysfunctions were included in logistic regression models for categorical variables and linear regression models for continuous variables. All analysis was two-tailed and the level of significance was set at *p* < 0.05.

### Ethical considerations

Ethical approval was obtained from the University of Ibadan/University College Hospital’s Ethics committee, reference number: UI/EC/16/0144. The purpose of the study was explained to all the participants and those who were willing to participate in the study provided written informed consent. Serial numbers were allotted to the participants to anonymise the questionnaires, such that data collected could not be linked to participants. Refusal to participate in the study did not alter the quality of treatments participants received.

## Results

### Socio-demographic and clinical characteristics of participants

Over the study period, 238 participants were eligible for inclusion in the study, of which 144 (60.5%) were male participants. The mean age of the participants was 35.71 years (standard deviation: 9.53 years). Over half of the participants had tertiary education (57.1%) and were single (55%), while 33.6% were unemployed and 39.1% were involved in unskilled work (such as causal factory workers, messengers, janitors and desk clerks).

Over three-quarters (78.6%) of the participants were aged above 18 years at the onset of illness, with a mean age of 25 years. About two-thirds of the participants (61.3%) presented for treatment within 1 year of onset of symptoms of their mental disorders and 63.4% had three or more previous episodes of mental disorders. Over half (54.2%) had been using medications for at least 12 months. In terms of diagnosis, the most common disorder presenting to this clinic was schizophrenia (61.3%), followed by bipolar affective disorder (16.8%) and depressive disorder (13%). Using the Beck’s Depression Inventory, regardless of diagnosis, 34.6% of the participants were found to have depressive symptoms, while low medication adherence was reported in 41.8% of patients (Table 1).

**TABLE 1:** Socio-demographic and clinical characteristics of participants.

Variables	Male		Female		Total	
	*n*	*%*	*n*	*%*	*n*	*%*
			
Total	144	60.5	94	39.5	238	100
**Age (years)**						
18–29	39	27.1	29	30.9	68	28.6
30–39	56	36.9	35	37.2	91	38.2
40–49	34	23.6	21	22.3	55	23.1
50–60	15	10.4	9	9.6	24	10.1
**Highest level of education**						
Primary	3	2.1	5	5.3	8	3.4
Secondary	64	44.4	30	31.9	94	39.5
Tertiary	77	53.5	59	62.8	136	57.1
**Marital status**						
Single	98	68.1	33	35.1	131	55.0
Married	41	28.5	51	54.3	92	38.7
Divorced/separated/widowed	5	3.5	10	10.6	15	6.3
**Religion**						
Christian	111	77.1	70	74.5	181	76.1
Islam	33	22.9	24	25.5	57	23.9
**Employment status**						
Skilled	36	25.3	29	30.9	65	27.3
Unskilled	60	41.7	33	35.1	93	39.1
Unemployed	48	33.0	32	34.0	80	33.6
**Age at onset of illness (years)**						
*≤* 18	25	17.4	26	27.7	51	21.4
Above 18	119	82.6	68	72.3	187	78.6
**Duration of illness before presentation (years)**						
*≤* 1	89	61.8	57	60.6	146	61.3
1–5	41	28.5	18	19.1	59	24.8
Above 5	14	9.7	19	20.2	33	13.9
**Number of previous episodes**						
1	23	16.0	11	11.7	34	14.3
2	33	22.9	20	21.3	53	22.3
3 or more	88	61.1	63	67.0	151	63.4
**Clinical diagnosis**						
Depression	16	11.1	15	16.0	31	13.0
Bipolar affective disorder	20	13.9	20	21.3	40	16.8
Schizophrenia	93	64.6	53	56.4	146	61.3
Others	15	10.4	6	6.4	21	8.8
**Presence of medical comorbidity**						
Yes	14	9.7	6	6.4	20	8.4
No	130	90.3	88	93.6	218	91.6
**Comorbid substance use**						
Yes	14	9.7	1	1.1	15	6.3
No	130	90.3	93	98.9	223	93.7
**Type of substance use (*N* = 15)**						
Alcohol	2	14.3	0	0.0	2	13.3
Cigarette	2	14.3	0	0.0	2	13.3
Alcohol & cigarette	4	28.6	1	100.0	5	33.3
Cannabis	6	42.8	0	0.0	6	40.0
**Medication**						
Atypical antipsychotics	73	50.7	39	41.5	112	47.1
Typical antipsychotics	41	28.5	27	28.7	68	28.6
Tricyclic antidepressants	10	6.9	8	8.5	18	7.6
Mood stabilizers	20	13.9	20	21.3	40	16.8
**Duration of use of psychotropic medications**						
*≤* 5 years	73	50.7	56	59.6	129	54.2
6–10 years	43	29.9	20	21.3	63	26.5
> 10 years	28	19.4	18	19.1	46	19.3
**Medication adherence**†						
Low	57	39.9	42	44.7	99	41.8
Medium	72	50.3	45	47.9	117	49.4
High	14	9.8	7	7.4	21	8.9
**Depressive symptoms**†						
Absent	100	69.4	55	59.1	155	65.4
Present	44	30.6	38	40.9	82	34.6

† , *n* < 144 among male participants and 94 among female participants because of missing data.

### Prevalence of sexual dysfunction

The overall prevalence of any sexual dysfunction among male participants was 84.7%, while this was 95.7% among female participants. The prevalence was significantly higher among female participants (*x*^2^ = 7.1, *p* = 0.008). The most common types of sexual dysfunction were sexual desire disorder, intercourse dissatisfaction and orgasmic dysfunction for male participants.

### Correlates of sexual dysfunction

The socio-demographic and clinical variables significantly associated with overall sexual dysfunction and the types of sexual dysfunction in male participants are shown in Table 2, while those for the female participants are shown in Tables 3 and 4. In male participants, overall sexual dysfunction, erectile dysfunction and sexual desire disorder were significantly associated with employment status (with highest rates among those who are unemployed), while orgasmic dysfunction was significantly associated with marital status (with highest rates among the divorced, separated and widowed).

**TABLE 2:** Socio-demographic and clinical correlates of sexual dysfunction among male participants.

Variable	Erectile dysfunction (106 [73.6%])		Orgasmic dysfunction (113 [78.5%])		Sexual desire disorder (125 [86.8])		Intercourse dissatisfaction (124 [86.1])		Overall dissatisfaction (110 [86.1])		Overall sexual dysfunction (122 [84.7])	
	*X*^2^	*p*-value	*X*^2^	*p*-value	*X*^2^	*p*-value	*X*^2^	*p*-value	*X*^2^	*p*-value	*X*^2^	*p*-value
**Age (years)**												
18–29	0.899	0.826	1.742	0.628	1.642	0.650	1.709	0.635	3.124	0.373	3.327	0.344
30–39	-	-	-	-	-	-	-	-	-	-	-	-
40–49	-	-	-	-	-	-	-	-	-	-	-	-
50–60	-	-	-	-	-	-	-	-	-	-	-	-
**Highest level of education**												
Primary	0.550	0.759	2.469	0.291	1.125	0.570	2.761	0.251	4.486	0.096	1.424	0.491
Secondary	-	-	-	-	-	-	-	-	-	-	-	-
Tertiary	-	-	-	-	-	-	-	-	-	-	-	-
**Marital status**												
Single	3.083	0.214	6.250	0.044*	0.835	0.659	0.267	0.875	4.211	0.122	1.544	0.462
Married	-		-		-		-		-		-	
Divorced/separated/widowed	-		-		-		-		-		-	
**Religion**												
Christian	1.484	0.223	0.284	0.594	0.043	0.836	0.112	0.738	0.070	0.791	0.330	0.566
Islam	-	-	-	-	-	-	-	-	-	-	-	-
**Employment status**												
Skilled	6.721	0.035*	2.491	0.288	12.733	0.002*	3.345	0.188	1.790	0.408	12.147	0.002**
Unskilled	-	-	-	-	-	-	-	-	-	-	-	-
Unemployed	-	-	-	-	-	-	-	-	-	-	-	-
**Age of onset of disease (years)**												
*≤* 18	0.089	0.766	1.626	0.202	0.038	0.846	0.877	0.349	0.003	0.960	1.238	0.266
Above 18	-	-	-	-	-	-	-	-	-	-	-	-
**Duration of illness before presentation (years)**												
*≤* 1	0.134	0.935	0.511	0.924	2.181;	0.336	3.291	0.193	0.802	0.670	0.453	0.797
1–5	-	-	-	-	-	-	-	-	-	-	-	-
> 5	-	-	-	-	-	-	-	-	-	-	-	-
**Number of previous episodes**												
1	4.555	0.103	2.962	0.227	1.873;	0.392	2.140	0.343	0.328	0.849	3.048	0.218
2	-	-	-	-	-	-	-	-	-	-	-	-
≥ 3	-	-	-	-	-	-	-	-	-	-	-	-
**Clinical diagnosis**												
Depression	2.520	0.472	3.295	0.348	4.080	0.253	4.987	0.173	6.017	0.111	1.319	0.725
Bipolar affective disorder	-	-	-	-	-	-	-	-	-	-	-	-
Schizophrenia	-	-	-	-	-	-	-	-	-	-	-	-
Others	-	-	-	-	-	-	-	-	-	-	-	-
**Presence of any comorbidity**												-
Yes	0.196	0.658	0.00	0.992	2.357	0.216	0.590	0.442	0.121	0.728	0.793	0.373
No	-	-	-	-	-	-	-	-	-	-	-	-
**Comorbidity substance use**												
Yes	0.694	0.405	1.847	0.174	0.918	0.338	0.737	0.391	1.260	0.262	0.012	0.914
No	-	-	-	-	-	-	-	-	-	-	-	-
**Duration of use of psychotropic medications**												
< 5 years	1.590	0.212	1.223	0.301	0.848	0.433	1.550	0.220	0.379	0.686	1.154	0.322
6–10 years	-	-	-	-	-	-	-	-	-	-	-	-
> 10 years	-	-	-	-	-	-	-	-	-	-	-	-
**Medication adherence**												
Low	0.408	0.666	1.579	0.212	1.267	0.287	3.374	0.039*	0.850	0.431	1.042	0.357
Medium	-	-	-	-	-	-	-	-	-	-	-	-
High	-	-	-	-	-	-	-	-	-	-	-	-
**Depressive symptoms**												
Absent	0.001	0.984	0.378	0.540	0.126	0.723	0.080	0.778	2.305	0.132	0.375	0.542
Present	-	-	-	-	-	-	-	-	-	-	-	-

* , Statistically significant at *p* < 0.05.

** , Remained statistically significant after logistic regression analysis.

**TABLE 3:** Socio-demographic and clinical correlates of overall sexual dysfunction among female participants.

Socio-demographic and clinical variables	Overall sexual dysfunction					
	Yes		No		*X*^2^	*p*
	*n*	%	*n*	%		
**Age (years)**						
18–29	28	96.6	1	3.4	1.014	0.798
30–39	33	97.1	1	2.9	-	-
40–49	20	95.2	1	4.8	-	-
50–60	9	90.0	1	10.0	-	-
**Highest level of education**						
Primary	5	83.3	1	16.7	7.530	0.023*
Secondary	26	89.7	3	10.3	-	-
Tertiary	59	100.0	0	0.0	-	-
**Marital status**						
Single	33	100.0	0	0.0	3.676	0.159
Married	46	92.0	4	8.0	-	-
Divorced/separated/widowed	11	100.0	0	0.0	-	-
**Religion**						
Christian	70	98.6	1	1.4	5.772	0.016*
Islam	20	87.0	3	13.0	-	-
**Employment status**						
Skilled	28	96.6	1	3.4	0.4107	0.814
Unskilled	31	93.9	2	6.1	-	-
Unemployed	31	96.9	1	3.1	-	-
**Age at onset of disease (years)**						
≤ 18	25	100.0	0	0.0	1.514	0.219
Above 18	65	94.2	4	5.8	-	-
**Duration of illness before presentation**						
≤ 1 year	53	94.6	3	5.4	1.130	0.568
1–5 years	17	94.4	1	5.6	-	-
Above 5 years	20	100.0	0	0.0	-	-
**Number of previous episodes**						
1	10	90.9	1	9.1	1.616	0.446
2	21	100.0	0	0.0	-	-
3 or more	59	95.2	3	4.8	-	-
**Clinical diagnosis**						
Depression	14	93.3	1	6.7	2.664	0.446
Bipolar affective disorder	19	95.0	1	5.0	-	-
Schizophrenia	51	98.1	1	1.9	-	-
Others	6	85.7	1	14.3	-	-
**Presence of any comorbidity**						
Yes	6	85.7	1	14.3	1.868	0.172
No	84	96.6	3	3.4	-	-
**Comorbidity substance use**						
Yes	2	100.0	0	0.0	0.090	0.763
No	88	95.7	4	4.3	-	-
**Duration of use of psychotropic medications**						
≤ 5 years	57	98.3	1	1.7	3.312	0.191
6–10 years	18	94.7	1	5.3	-	-
> 10 years	15	88.2	2	11.8	-	-
**Medication adherence**						
Low	40	95.2	2	4.8	1.786	0.409
Medium	43	97.7	1	2.3	-	-
High	7	87.5	1	12.5	-	-
**Depressive symptoms**†						
Absent	52	94.5	3	5.5	0.435	0.509
Present	37	97.4	1	2.6	-	-

* , Statistically significant at *p* < 0.05.

† , *n* < 94 among female participants because of missing data.

**TABLE 4:** Socio-demographic and clinical correlates of the domains of sexual dysfunction among female participants.

Socio-demographic and clinical variables	Sexual desire disorder		Arousal disorder		Lubrication disorder		Orgasmic dysfunction		Sexual dissatisfaction		Sexual pain disorder	
	*t/f*-test	*p*-value	*t/f*-test	*p*-value	*t/f*-test	*p*-value	*t/f*-test	*p*-value	*t/f*-test	*p*-value	*t/f*-test	*p*-value
**Age (years)**												
18–29	1.486	0.224	1.980	0.123	1.603	0.194	2.089	0.107	1.084	0.360	3.865	0.012*
30–39	-	-	-	-	-	-	-	-	-	-	-	-
40–49	-	-	-	-	-	-	-	-	-	-	-	-
50–60	-	-	-	-	-	-	-	-	-	-	-	-
**Highest level of education**												
Primary	1.078	0.345	2.328	0.103	3.941	0.023*	5.228	0.007*	0.715	0.492	2.754	0.069
Secondary	-	-	-	-	-	-	-	-	-	-	-	-
Tertiary	-	-	-	-	-	-	-	-	-	-	-	-
**Marital status**												
Single	1.180	0.312	10.325	0.001*	13.801	0.001*	12.155	0.001*	3.250	0.430	10.405	0.001*
Married	-	-	-	-	-	-	-	-	-	-	-	-
Divorced/separated/widowed	-	-	-	-	-	-	-	-	-	-	-	-
**Religion**												
Christian	−1.133	0.260	−2.430	0.017**	−2.167	0.033**	−2.103	0.038*	−1.487	0.140	−0.840	0.403
Islam	-	-	-	-	-	-	-	-	-	-	-	-
**Employment status**												
Skilled	0.082	0.921	3.840	0.025**	2.403	0.096	2.873	0.062	2.042	0.136	4.423	0.015**
Unskilled	-	-	-	-	-	-	-	-	-	-	-	-
Unemployed	-	-	-	-	-	-	-	-	-	-	-	-
**Age of onset of disease (years)**												
< 18	−2.235	0.028*	−0.803	0.425	−1.203	0.232	−0.918	0.361	0.255	0.800	−0.892	0.375
Above 18	-	-	-	-	-	-	-	-	-	-	-	-
**Duration of illness before presentation**												
< 1 year	1.380	0.257	0.213	0.808	0.058	0.944	0.110	0.912	0.076	0.940	0.329	0.743
1–5 years	-	-	-	-	-	-	-	-	-	-	-	-
Above 5 years	-	-	-	-	-	-	-	-	-	-	-	-
**Number of previous episodes**												
1	2.000	0.141	1.798	0.172	1.858	0.162	2.467	0.091	0.985	0.377	0.842	0.434
2	-	-	-	-	-	-	-	-	-	-	-	-
3 or more	-	-	-	-	-	-	-	-	-	-	-	-
**Clinical diagnosis**												
Depression	0.070	0.976	2.967	0.036*	2.931	0.026*	1.970	0124	1.064	0.368	0.803	0.495
Bipolar affective disorder	-	-	-	-	-	-	-	-	-	-	-	-
Schizophrenia	-	-	-	-	-	-	-	-	-	-	-	-
Others	-	-	-	-	-	-	-	-	-	-	-	-
**Presence of any comorbidity**												
Yes	0.081	0.936	0.208	0.835	0.699	0.486	0.201	0.841	0.697	0.488	1.070	0.288
No	-	-	-	-	-	-	-	-	-	-	-	-
**Comorbidity substance use**												
Yes	−1.872	0.065	−0.899	0.371	−0.481	0.632	−0.457	0.649	−0.883	0.380	−1.860	0.853
No	-		-	-	-	-	-	-	-	-	-	-
**Duration of use of psychotropic medications**												
≤ 5	1.590	0.212	1.223	0.301	0.848	0.433	1.550	0.220	0.379	0.686	1.154	0.322
6–10	-	-	-	-	-	-	-	-	-	-	-	-
> 10	-	-	-	-	-	-	-	-	-	-	-	-
**Medication adherence**												
Low	0.408	0.666	1.579	0.212	1.267	0.287	3.374	0.039*	0.850	0.431	1.042	0.357
Medium	-	-	-	-	-	-	-	-	-	-	-	-
High	-	-	-	-	-	-	-	-	-	-	-	-
**Depressive symptoms**												
Absent	0.001	0.984	0.378	0.540	0.126	0.723	0.080	0.778	2.305	0.132	0.375	0.542
Present	-	-	-	-	-	-	-	-	-	-	-	-

* , Statistically significant at *p* < 0.05.

** , Remained statistically significant after logistic regression analysis.

Autonomic side effect of psychotropic medications was significantly associated with the presence of sexual dysfunction in male participants (*t*_test_ = –2.007, *p* = 0.047) but not in female participants.

In the female participants, overall sexual dysfunction was significantly associated with educational status (*x*^2^ = 7.5; *p* = 0.02) and religion (*x*^2^ = 5.8; *p* = 0.02) (Table 3).

Disorder of sexual desire was associated with age of onset of mental disorders (with mean FSFI scores being lower among those who had age of onset before 18 years). Disorder of sexual arousal was associated with marital status (with mean FSFI scores lowest among the divorced, separated and widowed) compared to those who were married. Other socio-demographic associations for disorder of sexual arousal include religion, employment status (with mean scores lowest among those who are unemployed) and clinical diagnosis (mean scores were lowest among those with schizophrenia). Disorder of lubrication, on the other hand, was associated with educational status, marital status (mean scores lowest among the divorced, separated and widowed), religion and clinical diagnosis (with mean scores lowest among those with schizophrenia).

Orgasmic disorder was associated with educational status, marital status, religion and medication adherence (Table 4). Sexual pain disorder was associated with age (the scores being lowest in the 18–29 age group), marital status and unemployment status.

In both male and female participants, the presence or absence of depressive symptoms was not significantly associated with overall sexual dysfunction or any of the domains of sexual dysfunction.

These associations were explored further in multivariate logistic regression analysis in the male participants and linear regression models in the female participants. In the male participants, being unemployed (odds ratio [OR] = 5.4, confidence interval [CI] = 1.525, 19.259), or being employed in unskilled work (OR = 4.7, CI = 1.515, 14.279) and the presence of autonomic side effects of psychotropic medications (OR = 1.3, CI = 1.103, 1.620) were the significant predictors of overall sexual dysfunction. For the female participants, practising Christianity was a significant predictor of sexual arousal disorder (*B* = 1.092, CI = 0.252, 1.932) and lubrication disorder (*B* = 1.107, CI = 0.056, 2.158), while unemployment was a significant predictor of arousal disorder (*B* = –0.634, CI = –1.095, –0.173) and sexual pain disorder (*B* = –0.691, CI = –1.319, –0.063).

## Discussion

This study examined the prevalence and correlates of sexual dysfunction among male and female patients with mental disorders attending the medical outpatient department of the University College Hospital, Ibadan. Overall, about 85% and 96% of male and female participants, respectively, had at least one form of sexual dysfunction. In the male patients, the most common forms were disorder of sexual desire, orgasmic dysfunction and sexual dissatisfaction. Unemployment, not being in a marital relationship and autonomic side effects of medications were the most important correlates of sexual dysfunction in the male participants. While for the female participants, important correlates included religion, unemployment, marital status, early age of onset of mental disorder and having a diagnosis of schizophrenia.

These prevalence rates for sexual dysfunction in this sample of patients with mental disorders are much higher compared to rates obtained in studies of the general population that ranged between 30% and 81%.^3,4,5,6,7,8^ Hence, this finding further supports previous studies that have similarly reported higher rates of sexual dysfunction among patients with mental disorders, with reported rates ranging between 40% and 96%.^9,10,11,12,13,14,15,16,17,18,19^ When disaggregated by gender, earlier studies have reported rates similar to that of this study (75% – 83% in male participants and 82% – 96% in female participants).^10,11,15,16,19^ It is, however, pertinent to note that not all studies support the finding that patients with mental disorders have higher rates of sexual dysfunction compared to the general population.

Some studies have reported rates ranging between 40.4% and 68%, which is within the range reported in studies of general adult population.^9,12,14,18^ Factors that can account for the reported differences in rates include eligibility criteria for the selection of study participants and questionnaires used. In some of the studies that reported relatively lower rates, patients with comorbid psychoactive substance use disorder and comorbid medical conditions such as hypertension, diabetes, vascular diseases and other endocrine disorders were excluded.^9,12,14,18^ It is well known that these conditions commonly coexist with mental disorders and are established risk factors of sexual dysfunction; hence, excluding such patients generates lower rates and makes the sample unrepresentative and findings of such studies are not generalisable to regular patients with mental disorders.^2,31^

In this study, male participants who are either unemployed or unskilled labourers had up to five times the risk of sexual dysfunction compared to those who were employed. Employment status in men is reported in the literature as an important determinant of overall sexual dysfunction, erectile dysfunction and sexual desire disorder.^12,14^

One of the social roles conferred on the male gender is the ability to provide for the basic needs of his partner and this role may be more challenging when the man is not employed. Failure to achieve this leads to role reversal within a relationship contrary to societal and cultural expectations, thereby engendering feelings of shame and inadequacy.^12^ Furthermore, unemployment in a person with a mental disorder has been associated with public and self-stigmatisation which may cause low self-esteem and poor sexual performance or satisfaction.^32^

Another correlate of sexual dysfunction reported in male participants in this study is the presence of autonomic side effects of psychotropic medications as indicated by the significantly higher mean scores on the UKU side effect rating. While we could not ascertain the reason for this relationship in male participants, difference in dosing of psychotropic medications between male participants and female participants is a possibility. However, this present report is based on self-reported measures and we did not verify medication dose; hence, we could not explore this association. An earlier study by Smith et al.^18^ similarly found that sexual dysfunction was associated with autonomic side effects of psychotropic medications in male patients. However, that study went further to show that this association was related to plasma prolactin levels; sexual dysfunction was associated with the presence of autonomic side effects of medication in those with normal plasma prolactin levels, but this association ceased to exist in those with elevated prolactin levels. Measurement of plasma prolactin levels was beyond the scope of this study, and therefore we cannot state whether plasma prolactin level had any relationship with this finding; however, it is worth noting that when we controlled for other associated factors, autonomic side effects remained a significant risk factor for sexual dysfunction. Autonomic side effects were only significantly related to male sexual dysfunction and not female in this study. Acetylcholine, a major neurotransmitter in the autonomic nervous system, plays a vital role in male sexual function causing the relaxation of smooth muscles of the corpus cavernosum that, in turn, leads to vasocongestion and penile erection. Hence, anticholinergic activities from the side effects of psychotropic medications could result in erectile dysfunction in male patients. It is interesting that medication adherence was not a correlate of sexual dysfunction in male participants but was associated with orgasmic dysfunction in female participants.

Marital status was significantly associated with sexual dysfunction in both male participants and female participants in this study. Male participants who were divorced, separated, widowed or single were more likely to experience arousal disorder, while female participants who were divorced, separated and widowed were more likely to experience arousal, lubrication, orgasmic and sexual pain disorders compared to those who were married. This is consistent with the findings of a study by Fanta et al.^16^ who reported that being unmarried, divorced or widowed was significantly associated with sexual dysfunction among female patients with mental disorders. The functional impairment from mental disorders as well as the low sexual desire which is more common among female patients might have negatively affected interpersonal and social skills necessary to initiate and maintain a marriage relationship, hence worsening the experience of desired sexual life. Educational status was significantly associated with the overall sexual dysfunction, lubrication and orgasmic disorders. A few studies among patients with mental disorders have reported an association between sexual dysfunction and educational status,^33,34^ consistent with the findings of this study.

Female sexual dysfunction was related to age, age at onset of mental illness, clinical diagnosis and medication adherence. Although studies have found that sexual dysfunction including sexual pain disorder increases with age, this study found significantly lower mean scores for sexual pain disorder in female participants below the age of 30 years. While the specific reasons for this association in this population with mental disorders are unknown, young women in the general population have been reported to experience pain during sexual intercourse because of physical and psychosocial factors including congenital vulvovaginal abnormality, vulvovaginal infection, poorly repaired episiotomy after childbirth, pelvic trauma or surgery, sexual abuse and anxiety.^35,36,37^ Early age of onset of mental illness was found to be associated with disorder of sexual desire. The onset of mental illness in childhood and adolescence has been associated with greater illness severity, persistence of symptoms and lack of treatment response, which might affect the psychosexual development and adjustment essential for normal sexual functioning.^38^ Female patients with schizophrenia were found to be more likely to have sexual dysfunction in this study, which is similar to the findings of an earlier study among patients with schizophrenia in the United Kingdom.^10^

Religion and employment status emerged as the independent predictors of sexual dysfunction among female participants. Although religion has not been previously reported to be associated with sexual dysfunction in patients with mental disorders, religiosity has been reported to be a risk factor of sexual dysfunction in a community sample of women.^39^ It could be hypothesised that the association reported in this study may be because of the tendencies of some religious sects to consider the discussion of sexuality as a sacrilegious act, hence forbidding people, especially women, from talking about their sexual experiences and difficulties.^40^

The findings of this study should be interpreted bearing in mind the limitations. Firstly, this study employed a cross-sectional study design that makes the inference of the direction of causality between sexual dysfunction and the socio-demographic and clinical variables difficult. Secondly, the study was conducted in a tertiary facility where the patients may not be a true representation of the demographics in primary and secondary healthcare facilities. This is because patients who are better educated or from higher socio-economic classes may be over-represented in the population of patients receiving care in tertiary healthcare centres in Nigeria. Thirdly, the inclusion of patients with comorbid medical disorders as well as comorbid psychoactive substance use disorders could be a confounding factor as these disorders have been reported to also cause sexual dysfunction. There was also the possibility that some participants in this study had difficulty recalling events, such as age at onset of illness, duration of illness before presentation, duration of use of psychotropic medications, doses of medications and the number of previous episodes, which could have caused recall bias and affected the inference made. Furthermore, confounding factors that could have influenced the associations reported, such as the presence of comorbid medical disorders and hormonal levels, were not explored. Even though the instruments used in assessing sexual dysfunctions (the IIEF for male participants and the FSFI for female participants) have been assessed to have good psychometric properties in the Nigerian population, we cannot overlook the limitations of these instruments. These include their inability to measure distress and provide information about relevant aetiologic factors.

Despite these limitations, this study has contributed to expanding the knowledge base about the correlates of sexual dysfunction in male participants and female participants with serious mental disorders on treatment in Nigeria. The rates of sexual dysfunction were quite high among patients of both sexes with mental disorders. Although unemployment was a risk factor of sexual dysfunction in both male and female participants in this study, other factors were more specific to certain gender and type of sexual dysfunction. Medication adherence and depressive symptoms were not risk factors of sexual dysfunction among the participants, but autonomic side effect of psychotropic medications was a risk factor among male participants. While we did not explore the number of patients who were aware of this problem and the rates among those who had actually complained to their doctors, we know that this will be very low. There is an urgent need to increase the awareness about the high rates of sexual dysfunction in patients with mental disorders to encourage healthcare providers to routinely screen for them and provide appropriate interventions. More studies are required to further explore these associations with a view to determine the direction of causality and to explore the impact of sexual dysfunction on patient functioning and QoL.

Sexual dysfunction was highly prevalent among patients with mental disorders, particularly among female patients. Unemployment was a significant risk factor of sexual dysfunction in both sexes. In addition, while the presence of autonomic side effects of psychotropic medications predicted sexual dysfunction in male patients, practising Christianity as a religion predicted female sexual dysfunction.
